# Nuclear Medicine in the Philippines: A Glance at the Past, a Gaze at the Present, and a Glimpse of the Future

**DOI:** 10.7508/aojnmb.2016.02.009

**Published:** 2016

**Authors:** Patricia A. Bautista, Teofilo O.L. San Luis

**Affiliations:** 1 Department of Nuclear Medicine & PET, St. Luke’s Medical Center, Bonifacio Global City, Taguig, Metro Manila, Philippines; 2 Past President, Philippine Society of Nuclear Medicine; Former Dean, St. Luke’s College of Medicine – William H. Quasha Memorial; Former Dean, Asian School of Nuclear Medicine

**Keywords:** History, Nuclear, medicine

## Abstract

While the introduction of radioactive tracers in the study of metabolic pathways has been well-documented in clinical thyroidology as early as 1924, the widespread utilization in other clinical specialties has been hampered by slow developments in radiation-detecting devices and in the production of appropriate radiopharmaceuticals, in addition to the morbid fear of radiation. In the Philippines, the first radioisotope laboratory was established in 1956. Ten years later, the Philippine Society of Nuclear Medicine was formed. Through the years, challenges were overcome, foundations were laid down, growth was encouraged, friendships with other organizations were built, adjustments were made, and rules were enforced. To date, there are approximately 58 nuclear medicine centers randomly distributed from north to south of the Philippines, 7 accredited nuclear medicine training institutions, 95 board-certified nuclear medicine physicians (a few of whom are also internationally recognized), and a regionally-indexed Philippine Journal of Nuclear Medicine. Qualifying examinations for technologists were also recently instated. International relations are constantly strengthened by sending trainees abroad and accepting foreign trainees here, as well as participating in conferences and other endeavors. While the cost of putting up nuclear medicine centers in the Philippines is still prohibitive, it should not pose too much of a constraint as there are foreign and local parties willing to help. With appropriate instrumentation, targeting radiopharmaceuticals and trained human resources, nuclear medicine can indeed contribute much to health care delivery.

## Introduction

Nuclear Medicine is perceived as a specialty with high establishment, and high maintenance-costs, with varying levels of government support and investment in infrastructure (1). Its reason of being is the use of radioactive substances localized in organs-systems for diagnosis or treatment of diseases. These radioactive substances can be detected by specialized equipment to produce images or data useful to guide further clinical decision-making; or are meant to stay in a particular organ to destroy it functionally if not structurally.

While its introduction in the study of metabolic pathways has been well-documented in clinical thyroidology as early as the 1920s (2) its widespread utilization in other clinical specialties has been hampered by slow developments in radiation-detecting devices and in the production of appropriate radiopharmaceuticals. The morbid fear of radiation (occasioned by the destructive use of atomic energy in warfare in 1945 in Hiroshima & Nagasaki; and the harm caused by unfortunate incidents in nuclear power plants in 1979 in Three-Mile Island, in 1986 in Chernobyl, and in 2011 in Fukushima) added together for a more circumspect use of nuclear energy in Medicine.

The three required components for the beneficial use of nuclear energy in Medicine include appropriate instrumentation, targeting radiopharmaceuticals and trained human resources. Absence of any of the constituent triad makes Nuclear Medicine unlikely to be able to contribute to health care delivery at all.

In the Philippines, Nuclear Medicine as a specialty started with the opening of the radioisotope laboratory at the Philippine General Hospital in 1956 under the leadership of Dr. Paulo C. Campos (3, 4). Nuclear medicine scientists, as they were initially called, consisted of radiologists, internists and pathologists who underwent either short preceptorship abroad or formal training in nuclear medicine at university medical centers (5). They used ^131^I HAS, ^51^Cr-tagged red blood cells, ^131^I-triolein-oleic acid and ^60^Co-vitamin B12 for diagnostic purposes and ^131^I and ^32^P for therapeutic purposes.

It was in 1966 when these Nuclear Medicine scientists formed among themselves the Philippine Society of Nuclear Medicine (PSNM) with Dr. Paterno C. Chikiamco serving as its first president. PSNM became the third nuclear medicine society in Asia, after South Korea and Japan. Even in its very early years, PSNM’s role in the region was already recognized as it became one of the founding members of the Asian Federation of Nuclear Medicine in 1969.

At that time, PSNM had already formed close ties with the then Philippine Atomic Energy Commission (until it was reorganized in 1986 to become the current Philippine Nuclear Research Institute or PNRI). The International Atomic Energy Agency (IAEA) played a key role in the human resource development of these pioneering Filipino physicians who would receive study grants to train in established Nuclear Medicine institutions abroad.

By the end of the first decade of the PSNM, a number of nuclear medicine centers had been established in Manila, all of which had rectilinear scanners as their main imaging equipment. It was the John F. Cotton Memorial Hospital that had installed the country’s first gamma camera (3, 4).

### Period of Growth

In 1976, Dr. Edmundo V. Villacorta pioneered the use of computers with the gamma camera (6). He, along with Dr. Juan F. Torres, Jr., established the first structured residency program at the Philippine Heart Center for Asia (5). In 1977, the PSNM became an official, registered organization and hosted the 2^nd^ Asia and Oceania Congress of Nuclear Medicine in November 1980. Despite the world in crisis, the PSNM managed to gather together participants from 24 countries, including Professor Henry N. Wagner, Jr. and Nobel Prize Laureate Dr. Rosalyn Yalow. This Congress gave a boost to the practice of nuclear medicine so that the year after, the PSNM saw the need to standardize it through the creation of the Philippine Specialty Board of Nuclear Medicine (PSBNM). Thus, in 1982, six examinees successfully passed the PSBNM certifying examinations. Blazing the trail for others to follow as certified Nuclear Medicine diplomates were Drs. Teofilo O.L. San Luis, Jr., Orestes P. Monzon, Evelyn G. Laureta, Consolacion O. Obmerga, Benigno L. Ong, & Emmanuel C. Limlingan (6).

Only graduates of these training centers were qualified to take the PSBNM examinations and their graduation rites were held within the annual conventions of PSNM (Table 1). The conferment of Diplomate status carried with it the title of DPSBNM after one’s name. It is to be noted that for hospitals to be accredited as training centers, they must possess the required imaging units and instrumentation, offer varied procedures for patient care, and are staffed by at least two PSNM Diplomates and Fellows who are further designated as training officers. The 3-year Nuclear Medicine Residency Program must incorporate the required number of scan readings and reporting, clinical and radiological rotations, attendance to required scientific activities, as well as production of scientific papers appropriate to the year level. Unfortunately, because of these requirements, these training centers are all located in Metro Manila although plans are being made for the opening of training centers in the Visayas and Mindanao as they are now being staffed by PSNM Diplomates and Fellows.

**Table 1:** Showing the list of institutions with residency/fellowship training programs that are currently accredited by the PSNM, the year their respective programs began, and the number of diplomates produced ever since. There are 8 diplomates who trained abroad and 2 who graduated from the Philippine General Hospital which no longer has an accredited training program

Accredited training institutions	Start of training	Diplomates produced
Philippine Heart Center	1979	24
Jose Reyes Memorial Medical Center	1987	14
Makati Medical Center	1991	3
University of Santo Tomas Hospital	1996	16
Cardinal Santos Medical Center	1999	7
St. Luke’s Medical Center – Quezon City	2001	21
St. Luke’s Medical Center – Global City	2013	None yet

### Period of Development

Setting the pace for nuclear medicine instrumentation and diagnostic services were Makati Medical Center which installed the first SPECT system in 1986 while Cebu Doctors Hospital in 1990 became the first nuclear medicine facility to be established outside Metro Manila. In 1993, St. Luke’s Medical Center operated the first bone densitometer. Despite the rapid increase in number of advanced camera systems and radioimmunoassay laboratories, nuclear medicine procedures for the thyroid still accounted for 2/3 of the imaging procedures done in the Philippines in contrast to oncologic and cardiovascular applications, which constituted the bulk in Western countries. Radioiodine therapy also comprised 99% of all radionuclide therapies, with only a few treatments done for bone pain, malignant effusions, and hemarthrosis (7).

Prior to 1993, the PSNM held its annual conventions in conjunction with those of the bigger organization Radioisotope Society of the Philippines (RSP) or with conferences of other related professional associations (7, 8). Thereafter, PSNM, despite being a small society, managed its own annual conventions. It even sponsored the organization of a number of nuclear medicine physicians and cardiologists into the Philippine Association of Nuclear Cardiology (PANC) under its umbrella. Also in 1994, PSNM launched “Images,” the official newsletter of the Society, and established the PSNM Section of Nuclear Technologists (9). Meanwhile, RSP has ceased to exist and in its place are now a number of radiation-related specialty societies of particular concerns, like the Philippine Association for Radiation Protection (PARP), Philippine Organization of Medical Physicists (POMP), Philippine Radiation Oncology Society (PROS), Philippine Society for Non-Destructive Testing (PSNDT), among others.

In 1996, the PSNM had two conventions, one incorporated in the International Congress of Internal Medicine (ICIM) and the other integrated in the 2^nd^ Philippine Nuclear Congress (PNC). In February 1996, the PSNM inducted its first two Fellows as still another and higher category of PSNM membership. After at least two years of practice, authorship of presented and published scientific papers, an exemplary moral behavior, and upon invitation by the PSNM Board of Directors, Diplomates are elevated to the rank of Fellows with the distinctive FPSNM proudly affixed to their names. It was also in that year when PSNM published and distributed the monograph “Nuclear Medicine in the Philippines 1995.”

### Period of Ascendancy

In 1999, Dr. Jerry M. Obaldo became the course director of the first IAEA Regional Training Course in the country and began the tradition of regularly having foreign speakers during annual conventions. He also represented the Philippines during the first working group meeting of the Asian Regional Cooperative Council for Nuclear Medicine (ARCCNM) in 2001 (8). In 2002, St. Luke’s Medical Center in Quezon City opened the first PET center in the Philippines and in Southeast Asia with Dr. Jonas Francisco Y. Santiago as its first director. In 2003, the same institution introduced transarterial rhenium lipiodol treatment for hepatocellular cancer through a grant from the IAEA.

Also in 2003, the PSNM released the first issue of the Philippine Journal of Nuclear Medicine (PJNM) with Dr. Obaldo as first editor. In 2004, St. Luke’s Medical Center acquired the first SPECT-CT camera. In recognition of the extraordinary contributions to the Society of Dr. Manfred Fischer of Germany and Dr. Ajit Padhy of IAEA, PSNM conferred on them the title of Honorary Fellow in the PSNM annual conventions of 2004 and 2005, respectively (8).

In 2006, the PSNM ratified a new constitution to adapt to the changing times. Also in 2006, St. Luke’s Medical Center performed the first radioimmunotherapy for lymphoma. In 2007, the PSNM co-hosted the 9^th^ Congress of the Asia and Oceania Thyroid Association. PSNM also held the first in-service examinations for residents of hospitals with accredited training programs. It was also the year when Dr. Emerita A. Barrenechea received the prestigious Jose Rizal Award from the Philippine Medical Association for her government service. During the ARCCNM Meeting 2007, and on the occasion of the 2^nd^ International Conference of Radiopharmaceutical Therapy (ICRT) held in Ulanbaator, Mongolia, Dr. Teofilo O.L. San Luis, Jr. was elected as Dean of the Asian School of Nuclear Medicine (ASNM) (and was reelected in the ARCCNM Meeting 2010 in Dhaka, Bangladesh).

In 2008, St. Luke’s Medical Center acquired the first PET-CT scanner. It was also during this year that PSNM attempted to have another newsletter, this time named “Scintillations.” The Society also launched the PSNM hymn. In 2009, the PSNM inducted its first foreign diplomates after having taken the required PSBNM examinations. In 2010, the Philippine Journal of Nuclear Medicine became included in the Western Pacific Region Index Medicus.

In 2013, PSNM cooperated with Dr. Richard Baum of Germany and the other members of the World Association of Radiopharmaceutical and Molecular Therapy (WARMTH) in successfully holding the 8^th^ International Conference on Radiopharmaceutical Therapy in Manila. In 2014, St. Luke’s Medical Center performed the first MIBG therapy.

Rising to the challenge of ARCCNM to develop new leaders in Nuclear Medicine for the Asian Region, some 27 young Nuclear Medicine physicians from all over Asia took the first-ever standardized written and oral examinations administered by the Asian Nuclear Medicine Board (ANMB) held in Osaka, Japan, in November 2014. Among them were Dr. Eduardo Erasto S. Ongkeko and Dr. Jefferson R. Pagsisihan who earned the title of “Fellow, Asian Nuclear Medicine Board” (FANMB). In November 2015, two more young Filipino physicians in the persons of Dr. Alvin P. Quiñon and Dr. Apolinario M. de Gracia, Jr. joined 49 other physicians in the ANMB exams given in Jeju, Korea and added the distinction of having FANMB after their names. The year 2015 saw its ending with the holding of the 3^rd^ Philippine Nuclear Congress with the participation of PSNM in its session on Health & Medicine on Day 2 of its 3-day congress, highlighting the contribution of radioiodine in hyperthyroidism and thyroid cancer and the role of Nuclear Medicine physicians in patient care.

### Period of Consolidation

**Figure 1:**
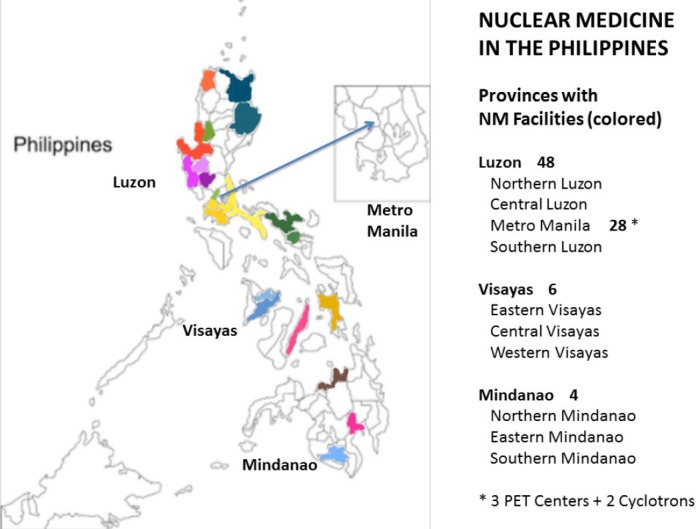
Showing the locations of the Nuclear Medicine centers currently operating in the Philippines. There are 48 in Luzon, 28 of which are in the National Capital Region, 6 in Visayas, and 4 in Mindanao. There are 1-4 isolation rooms per center for radioiodine therapy. Out of the 58 NM centers, all have radioimmunoassay laboratories, most have gamma cameras, while 7 offer SPECT-CT. The 3 PET Centers and 2 cyclotrons are located in the National Capital Region

The year 2016 started with the inauguration of the Philippines’ first-ever centralized cyclotron facility and PET/CT scanner at the National Kidney and Transplant Institute (NKTI) compound. K Health Corporation’s medical cyclotron, now fully operational, complements St. Luke’s Medical Center’s privately owned cyclotron, in meeting the demands for PET/CT imaging. These cyclotrons are now capable of supplying other Nuclear Medicine facilities with short-lived radiopharmaceuticals for advanced medical imaging services in oncology, neurology, urology, cardiology and pediatrics. The centralized cyclotron facility joins the PNRI’s Technetium production laboratory in offering radiopharmaceuticals required in routine clinical investigations. It is envisaged that more Nuclear Medicine centers will be established either with modest – or more ambitious – instrumentation according to local needs, and at a much lower cost. Already, more services are now available from Northern Luzon to Southern Mindanao with each center offering a wide array of diagnostic imaging and non-imaging services and a few therapeutic modalities. Most of the centers are concentrated in Metro Manila, which has a population of around 12 million out of the total 102 million Filipinos (Figure 1). The census varies in each center but the predominant procedures remain to be thyroid scans, bone scans, renal scans and myocardial perfusion scans.

Scanning the horizon for Nuclear Medicine, one can discern a significant growth in the specialty with more trainees coming in and more referrals for special procedures like radio-guided surgery and selective internal radiation therapy. As more and more conventions of PSNM and other specialty societies are being held outside Metro Manila, Nuclear Medicine rides on these interfaces with these societies to promote its widespread patronage and utilization (10). This augurs well for the practice of Nuclear Medicine as this will also provide job opportunities for the Diplomates and Fellows of PSNM. Currently, there are now 95 physicians fully-trained and certified to practice Nuclear Medicine in the Philippines. An even greater number of approximately 250 Nuclear Medicine technologists, medical physicists, radiochemists & radiopharmacists comprise the PSNM Technologists Section.

### Future Direction

While the cost of establishing Nuclear Medicine centers in the Philippines is still prohibitive, it should not pose too much of a constraint as there are joint ventures with interested foreign parties as well as local consortia willing to put up independent and free-standing units in progressive cities and municipalities with high patient throughput. It is envisaged that in the next 5-10 years there will be NM centers in all the remaining regions of the country. Distance to high quality medical care offering Nuclear Medicine services will be definitely shortened by these facilities manned by PSBNM-certified physicians and allied technical professionals.

The recently-installed mechanism for the certification of Nuclear Medicine Technologists through its own qualifying examinations administered by the PSNM Technologists’ Section raises the bar of competence among these technologists who can stand toe-to-toe with the counterparts even in more developed economies. The development of a special undergraduate curriculum leading to a Bachelor’s degree in Nuclear Medicine Technology (BSNMT), parallel to the long-established curricula of Medical Technology (BSMT) and Radiologic Technology (BSRT) offered in many colleges & universities is also a step in the right direction. This tract for technologists with major subjects in radiopharmacy and radiochemistry will enhance their competitiveness as allied medical professionals working with Nuclear Medicine physicians.

The Philippines is poised to be a training center for neighboring countries because of its well-structured Residency & Fellowship Training Programs suitable for application in most developing countries (11). The country is English-speaking and has become the favorite site for IAEA Regional Training Courses and international conferences of medical associations. A number of PSNM past presidents and other stalwarts have gone on Consultancy assignments and Experts’ Mission for the Agency assisting in the development of e-learning materials & curriculum, training trainors for NMDI’s QUANUM programmes, and other regional initiatives & projects to enhance patient care through clinical applications (12). One of PSNM’s Fellows is now working with IAEA Section of Nuclear Medicine & Diagnostic Imaging (NMDI) in the person of Dr. Thomas Neil B. Pascual. The Asian Nuclear Medicine Board (ANMB) itself has targeted the growing number of its Fellows who had taken its certifying examinations to assume greater leadership roles in the national and regional affairs and the Filipino physicians who have gotten on-board ANMB will undoubtedly take on these challenges.

The past officers and members of the PSNM have laid down a solid foundation for the next generation. In celebrating 50 years of the Society, their efforts must not be wasted. Values and lessons learned throughout the years must not be forgotten. Relationships among physicians, technologists, physicists, radiochemists, radiopharmacists and other allied professionals must be nurtured as Nuclear Medicine facilities are not places of earning a living but a nidus of professionals having families-away-from-home.
